# Carcinoma in situ of the pancreas with fibrosis area around the carcinoma: A case report

**DOI:** 10.1097/MD.0000000000022645

**Published:** 2020-10-16

**Authors:** Takeshi Mori, Atsushi Yamaguchi, Toshio Kuwai, Hirotaka Kouno, Noriaki Matsuura, Naoyuki Toyota, Shin Nakahira, Kazuya Kuraoka, Hiroshi Kohno

**Affiliations:** a Department of Gastroenterology; b Department of Radiology; c Department of Surgery; d Department of Pathology, National Hospital Organization Kure Medical Center and Chugoku Cancer Center, Kure, Hiroshima Prefecture, Japan.

**Keywords:** carcinoma in situ, case report, computed tomography, fibrosis, pancreas, pancreatic cancer, pancreatic intraepitherial neoplasia

## Abstract

**Rationale:**

Pancreatic cancer (PC) has the worst prognosis among all carcinomas. However, patients with carcinoma in situ (CIS) of the pancreas, usually, have a good prognosis. Many previous reports have mentioned the high frequency of fibrosis around CIS. In some cases, the fibrosis is detected on endoscopic ultrasonography (EUS), but there are few past reports of fibrosis detected on computed tomography (CT).

**Patient concerns:**

We encountered a case of fibrosis around CIS detected by CT. A 74-year-old man was being followed for chronic hepatitis C. On a contrast-enhanced CT (CE-CT), a space-occupied lesion (7 mm in size) in the pancreatic head was identified in the delayed phase.

**Diagnosis:**

It was shown to be a hypo echoic lesion in EUS, and EUS-fine-needle aspiration was performed. Cytological examination revealed abnormal cells suspicious for a neuroendocrine tumor.

**Interventions:**

Consequently, a pancreaticoduodenectomy was performed. Histopathological examination showed CIS in the branch duct with 10 mm of fibrosis around CIS. The fibrotic area corresponded to the mass detected by preoperative CE-CT.

**Outcomes:**

He had no relapse of PC but died 2 years later from another cause.

**Lessons:**

This case highlights the importance of identifying the enhanced area in the delayed phase on CE-CT, as this can be fibrosis around CIS.

## 1 Introduction

Pancreatic cancer (PC) is difficult to detect at an early stage, so most cases are diagnosed when locally advanced or in metastatic situations and thus prognosis is typically very poor. In contrast, carcinoma in situ (CIS) of the pancreas has a very good prognosis.^[1]^ CIS doesn’t form a mass, thus detection is difficult despite the recent development of computed tomography (CT), magnetic resonance pancreatocholangiography (MRCP), and endoscopic ultrasonography (EUS). Most cases of CIS are diagnosed by pancreatic juice cytology, and the motives for such tests are promoted by secondary changes, such as retention cysts, main pancreatic duct stenosis, and dilatation.^[2–4]^ Many past reports mentioned the high frequency of the existence of fibrosis around CIS.^[5–9]^ Further, fibrosis is detected as a low echo area in EUS.^[10]^ There are few past reports of the fibrosis being detected in contrast-enhanced CT (CE-CT). Thus, we report a case of the fibrosis around CIS detected by CE-CT. This observation may be an important clue to the detection of CIS.

## 2 Case report

**Figure 1:**
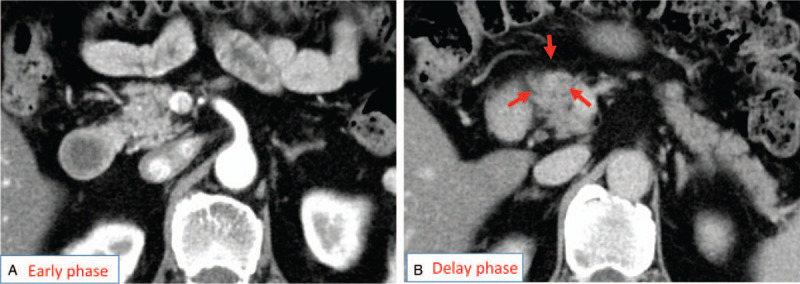
Abdominal computed tomography (dynamic study, sliced in 5 mm) revealed no abnormal mass in the early phase (A), but there was a weak enhanced round mass (arrows) detected in the pancreas head at the delay phase (B).

**Figure 2:**
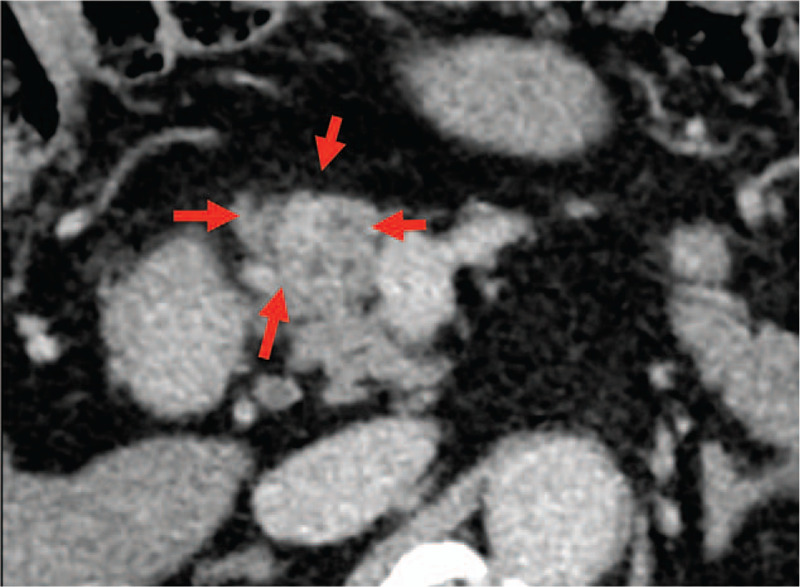
The pancreas mass was oval with irregular edges and the boundary was unclear in a 2 mm slice scan (arrows).

**Figure 3:**
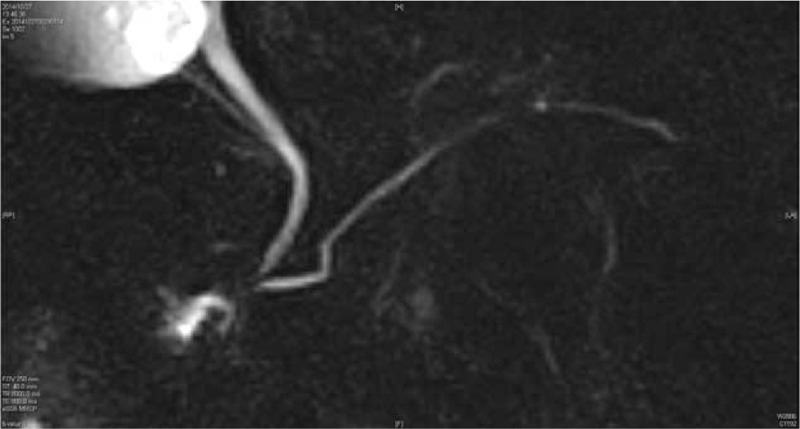
Magnetic resonance cholangiopancreatography did not reveal any abnormal changes in the pancreas head.

**Figure 4:**
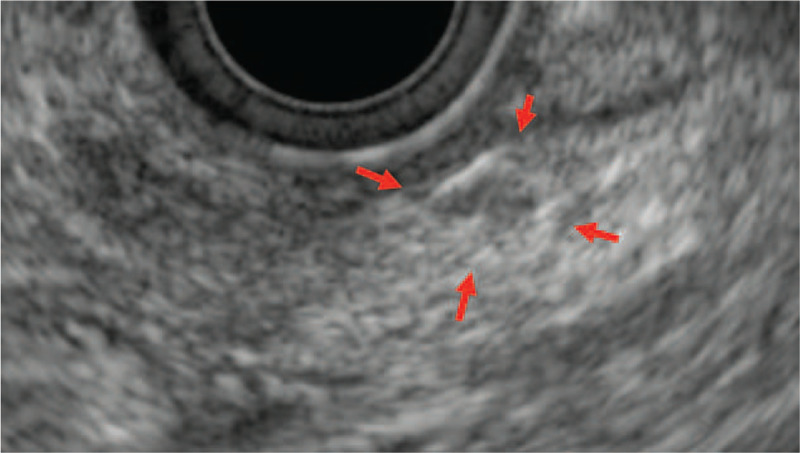
Endoscopic ultrasonography revealed a 6 mm nummular hypoechoic mass, the margin was relatively clear and smooth.

The patient was a 78-year-old man being followed for chronic hepatitis C virus infection at our hospital. An abdominal CE-CT had been performed annually. In September 2014, a CT scan revealed a 7 mm mass in the pancreatic head, which promoted further examination. The patient had a diabetes mellitus and chronic hepatitis, but no history of chronic pancreatitis. The patient didn’t smoke or drink alcohol, and had no notable family history including of pancreatic cancer. Physical examination results on admission were as follows: height, 167 cm; weight, 54 kg; and body temperature, 36.4 °C. His abdomen was soft and flat with no palpable mass. Relevant laboratory data were glutamic oxaloacetic transaminase 49 IU/L, glutamic pyruvic transaminase 62 IU/L, fasting blood sugar 250 mg/dL, hemoglobin A1c 8.9%. Relevant tumor markers and pancreatic enzymes were within normal ranges. Abdominal CT (in 5 mm slice scan) revealed no space occupied lesion (SOL) of the pancreas in the plain phase and early phase, but there was a 7 mm round enhanced SOL that was slightly detected in the head of the pancreas in the delayed phase (Fig. 1A and B). The tumor's margin was irregular and boundary was unclear in 2 mm slice scan (Fig. 2). EUS revealed a 6 mm nummular hypoechoic mass, the margin was unclear and not smooth (Fig. 3). MRCP revealed no cystic lesion or main pancreatic duct abnormality (Fig. 4). We conducted EUS fine needle aspiration (EUS-FNA), considering pancreatic cancer, mass forming pancreatitis, and a neuroendocrine tumor. Cytology showed small atypical cells suspicious for neuroendocrine tumor of the pancreas, therefore pancreaticoduodenectomy was performed.

**Figure 5:**
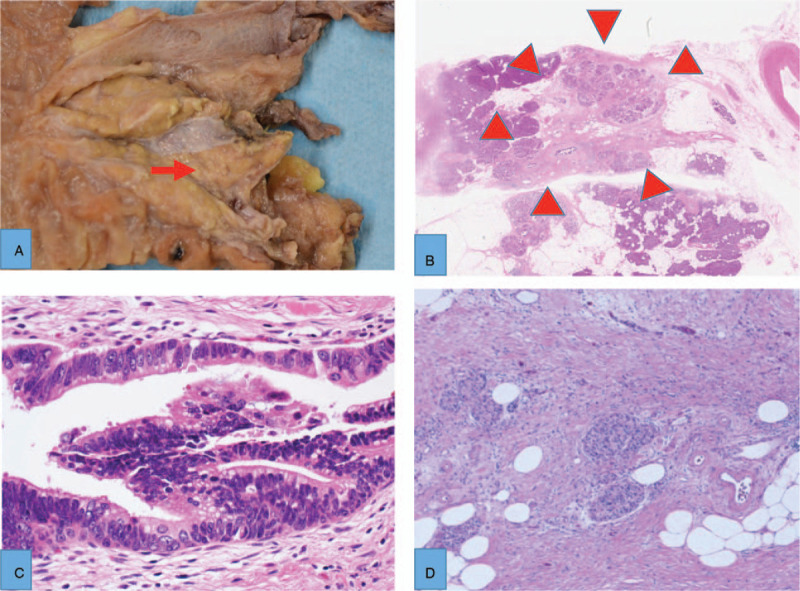
In gross findings, there was a white mass (arrow) at 10 mm (A) and the mass was histopathologically fibrosis (arrowheads) (B). In the fibrotic area, carcinoma in situ (C) had spread into the branch duct and there was hypertrophy of the neuroendocrine tissue (D).

Surgical findings were as follows. The pancreas was soft and normal. The pancreas head mass was detected in intraoperative US and surgery was done. Gross findings showed a slightly white mass consistent with fibrosis. Histopathological findings showed a 10 mm fibrotic lesion. This lesion included atrophic aciner cells and hypertrophic endocrine cells. Within this lesion, carcinoma in situ was located in the pancreatic branch duct (Fig. 5). Other sites were normal pancreas without inflammation and fibrosis. The final diagnosis was CIS in focal pancreatitis in the pancreas head 5 mm × 1 mm, Ph, pTS0, masked type, pTis, CH-, pS-, pRP-, pOO-, pPCM, pBCM, R0, ly0, v0, ne0. The atypical cells detected by preoperative EUS-FNA were thought to be hyperplastic neuroendocrine cells. He had no relapse of PC, but died in 2 years later because of another disease from PC.

The patient was scanned using a 128-detector CT instrument (Somatom Definition AS 128, Siemens Healthcare, Erlangen, Germany). Three-phase contrast-enhanced CT images of the pancreas were obtained during the early (40 seconds after contrast injection) and equilibrium phase (150 seconds after contrast injection). The contrast dose used was 600 mg/kg of the patient's body weight. We performed EUS and EUS-FNA using a GF-UCT260 convex echoendoscope (Olympus Corporation, Tokyo, Japan). The patient was scanned with a 1.5-Tesla MR scanner (Intera Achieva Pulsar, Philips Medical Systems, Best, The Netherlands) with a Sense body coil 4ch for three- and two-dimensional MRCP.

## 3 Discussion

Pancreatic ductal carcinoma is the most frequent PC, and also has the worst prognosis of all carcinomas. The 5-year survival rate is 7.1% in Japan.^[11]^ However, patients with Stage 0 (Union for International Cancer Control) PCs, so called, CIS actually have a good prognosis (85.8% with 5-year survival rate,^[11]^ 94.7% with 10-year survival rate^[12]^). Therefore, it is very challenging and worthwhile to diagnose CIS of the pancreas.

CIS of the pancreas is defined as a lesion localized in the pancreatic duct with flat or serrated papillary growth different from an intraductal papillary mucinous neoplasm.^[13]^ Hruban et al^[14]^ pioneered the concept of a “pancreatic intraepitherial neoplasia” (PanIN) in 2001 and classified PanINs for PanIN 1A, 1B, 2, and 3 according to the extent of the histological atypia. CIS is included in PanIN3 that have the severest atypia.^[14]^ PanINs are thought to be a precursor of invasive carcinoma because they always have abnormalities of genes that are often seen in invasive cancers.^[2,15]^ Moreover, operations in time of PanIN3 (CIS) stage should improve the prognosis of the patients with PC.^[12]^

As CISs don’t usually show any formed mass, they can’t be detected by any imaging examinations. Hence, the diagnosis of CISs is very difficult and most CISs are diagnosed with pancreatic juice cytology at endoscopic retrograde cholangiopancreatography (ERCP). The motives for pancreatic juice cytology are the existence of indirect abnormalities, such as re-tension cysts, main pancreatic duct dilatation, and/or stenosis. Some institutions in Japan have tried to diagnose CISs with various techniques. For instance, Iiboshi et al^[6]^ and Nakaizumi et al^[16]^ obtained successful results with serial pancreatic juice aspiration cytologic examination and secretin infusion at the time of ERCP, respectively. In contrast, EUS-FNA is still useless for diagnosis of CIS because CIS don’t form mass. Therefore, the example of CIS diagnosed with EUS-FNA is only one case^[17]^ of the 51 cases in PubMed and Igakuchuozassi (Japan) at June 2020.

Recently, there is interest in the fact that CISs frequently are accompanied by inflammation and surrounding fibrosis,^[2,7–9]^ and this characteristic is thought to be a clue for picking up CIS.^[18]^ The mechanism for forming fibrosis is thought to occur in 2 ways. First, CIS obstructs the flow of the branch duct and induces obstructive pancreatitis. Second, there is an immunological reaction to the cancer. In any way, this inflammatory change might induce main pancreatic duct stenosis and following duct dilatation of end side. In addition, these fibrotic areas might show a mild hypoechoic area in EUS, although they are not usually detected by CT, Magnetic resonance imaging, and abdominal ultrasonography.^[18]^

In our case, a fibrotic area around the CIS was confirmed pathologically, and was pointed out by EUS with a 6 mm hypo echoic mass before surgery. Furthermore, the most important consideration in our case was that the fibrosis was detected as a delayed enhancement in a dynamic CT study. Theoretically, CIS and associated fibrosis should be detectable because a fibrosis-rich lesion shows as iso or hypo dense in the early phase and high dense in delay phase (delayed enhancement) in CE-CT. We were able to catch mild change (small size and slightly enhancement). In this approach, searching for such fibrotic area with EUS and also CT might be useful for picking up CISs.

Another problem is that invasive cancer might have the same CT imaging features as fibrosis around CIS, because these 2 diseases are rich in a fibrotic component. Additionally, delayed enhancement in CT is also said to be a clue for invasive cancers. Empirically, however, the degree of enhancement is usually higher in invasive cancers than in our case. The fibrosis around CIS is difficult to point out in CT, so our case is very rare. Even in EUS, the change appeared as slightly hypoechoic compared with a normal pancreas.^[18]^ Given these findings, the degree of fibrosis might be weaker in CIS than in invasive carcinoma. Upon finding a fibrotic lesion in the pancreas, we should keep in mind both invasive carcinoma and also fibrosis around CIS. In addition, if the lesion can’t be diagnosed by EUS-FNA, we should consider performing pancreatic juice cytology for detecting CISs.

In conclusion, we have experienced a case of CIS around which a fibrotic area that showed delayed enhancement in dynamic CT. This change was very slight and difficult to point out, thus it is important to carefully consider such mild abnormalities in CT for the existence of fibrosis around CIS.

## Acknowledgments

The authors wish to thank Kanami Okino for helping collect the data and conduct the literature search.

## Author contributions

**Conceptualization:** Takeshi Mori, Atsushi Yamaguchi.

**Data curation:** Atsushi Yamaguchi.

**Interpretation of radiogram:** Noriaki Matsuura, Naoyuki Toyota.

**Pathological diagnosis:** Kazuya Kuraoka.

**Patient care:** Takeshi Mori, Atsushi Yamaguchi.

**Patient's surgeon:** Shin Nakahira.

**Writing – original draft:** Takeshi Mori and Atsushi Yamaguchi.

**Writing – review & editing:** Atsushi Yamaguchi, Toshio Kuwai, Hirotaka Kouno, Hiroshi Kohno.
